# Our Public Schools

**Published:** 1861-09

**Authors:** 


### OUR PUBLIC SCHOOLS

We certainly admire the thoroughness, promptness, and efficiency with which several of the public schools of New York City are conducted. With the girls' school in Twelfth-street, number forty-seven, and in Twentieth-street, number fifty, we are somewhat acquainted, and are persuaded that no one can spend an hour in either of them without being carried away with admiration and thankfulness, that such high privileges and opportunities are within the reach of every child in the city, costing nothing. To some of the features of the public schools there are strong objections. It is nothing short of a barbarism to keep children at study from nine until three, many of whom are but four or five years old. This enormity is palliated somewhat by recreations or bodily activities every forty-five minutes; still, it would be greatly better for children, under ten, not to be kept at study longer than two hours at a time, twice a day, and to have nothing at all to learn in the intervals of school time. Not only are they kept in six hours a day, but have such a variety and length of lessons to learn at home, that play or rest is out of the question, except between three and five o'clock, when it becomes too late to be out in winter; and in those two hours they have to come home and take their dinners, leaving in reality but a single hour out of the twenty-four for joyous out-door play. And when it is remembered that of a winter's morning, breakfast cannot be over sooner than eight o'clock, and at half past eight they must start for school, the conviction must force itself on the mind, that to some children, at least, it is a species of martyrdom. The true system is, let the children learn while they are in school some four hours a day; but when out of school, let not the hours of glorious play be half-blighted by constant thought of the unlearned task. But even here there is some apology for the course pursued. The unfortunate poor cannot afford to be without the services of their children later than twelve or fourteen, and all the education they ever get must be had before that time; hence they must be driven some. Under the circumstances, we advise those who are better off in the world, to discourage the "promotion" of their children, and by taking them from school about the first of June, allow their class to pass up higher, while they remain to go on in the regular line, with the long interval from June to September for a perfect *abandon* of recreation in the country

We earnestly trust that "reception days" and "public examination days" will be universally abolished; they are nothing but a sham; they are literally a "vain show;" they glorify the teachers at the sacrifice of the health and time and enjoyment of the children, who are unwholesomely stimulated, and to an extent sometimes which perils life itself.

As to the reading of the Bible in the public schools, there can be but one opinion in the mind of any man of common-sense and moderate intelligence, and that is, it ought not to be dispensed with, and ought to be enforced. But then there ought to be a just liberality in this regard, and it is suggested with considerable diffidence, that a spirit of accommodation be cultivated. In some respects the Protestant Bible differs from the Roman Catholic Bible. Let certain schools be set apart for the children whose parents prefer they should hear the Roman Catholic version. No Protestant would submit to have his children hear the Roman Catholic version by compulsion, nor should he wish to place his Roman Catholic fellow citizen in a position which he could not endure himself. Right-minded men can scarcely object to this modification, and we trust that something of the kind will be adopted speedily, in a generous, compromising spirit, and let all go on harmoniously in the common war against ignorance and degradation and unthrift.

In former years the public schools were patronized only by the acknowledged poor; but when their thoroughness and efficient administration became known to families of position, education, and wealth, they one by one began to lay aside their prejudices against conjectured contaminations arising from associations with the "vulgar herd," and became willing to risk them for the sake of the advantages of an impartial, systematic, and thorough mode of instruction; they could look under the surface and see that it was not without a benefit for their children to be thrown into circumstances where correct deportment and scholarship would entitle them to distinction, instead of family names, expensive dress, and useless ornaments. So that now the anomaly is seen of the children of the richest standing in the same class with those of the poorest, while the middle classes—those who have to rent their own house, who are trying to climb, or do not feel secure of their position—are afraid to send to the public schools, and are reduced to painful stintings and screwings to meet the tax of full two hundred dollars a year, as the cost of the privileges of a fashionable city school.

That the last year or two, especially of girls, might be profitably spent at the first-class private schools of the city, is not to be denied; but let the first years be spent at the public schools, and let the children of the rich, by their better conduct, bring the children of the poor up to them, and thus be to them an encouragement and a blessing; for a calico dress or a patched jacket full often covers a noble heart, and the chances are even that in a generation the calico and the patch will be "at the top of the heap."

